# Strategies for intranasal delivery of vaccines

**DOI:** 10.1007/s13346-012-0085-z

**Published:** 2012-07-12

**Authors:** Mehfuz Zaman, Saranya Chandrudu, Istvan Toth

**Affiliations:** 1 School of Chemistry and Molecular Biosciences (SCMB), The University of Queensland, St. Lucia, 4072 QLD Australia; 2 School of Pharmacy, The University of Queensland, Brisbane, QLD Australia

**Keywords:** Mucosal adjuvant, Nasal vaccine, Immunity, Vaccine delivery

## Abstract

The vast majority of human pathogens colonize and invade at the mucosal surfaces. Preventing infection at these sites via mucosally active vaccines is a promising and rational approach for vaccine development. However, it is only recently that the stimulation of local immunity at the mucosal surfaces has become a primary objective in addition to inducing systemic immunity. This review describes vaccine formulations designed for mucosal delivery to the nasal-associated lymphoid tissue, via intranasal administration. The association of antigens with mucosal adjuvants and delivery systems is emphasised.

## Introduction

Vaccination is one of the most successful accomplishments of medical science. Diseases that were prevalent are now increasingly rare because of the widespread use of vaccines. Next to clean water, no single intervention has had such an overwhelming effect on reducing mortality from childhood diseases as the use of vaccines [1]. The smallpox vaccine has eradicated a disease that had a 30 % fatality rate [2]. After over two decades of intensive efforts, the global polio eradication initiative is approaching its final stage aided by the two polio vaccines developed by Jonas Salk and Albert Sabin [3]. Nevertheless, next-generation vaccines are required to combat prevalent diseases.

An important field in the development of next-generation vaccines is the development of vaccines suitable for mucosal immunization. Most viral and bacterial infections start at the mucosal surfaces; thus, immunity against infective agents may depend on the induction of a mucosal immune response. As a result, for certain infectious agents, the mucosal route is the most appropriate method of immunization because it has been reported to induce both mucosal and systemic immune responses [4, 5].

**Fig. 1:**
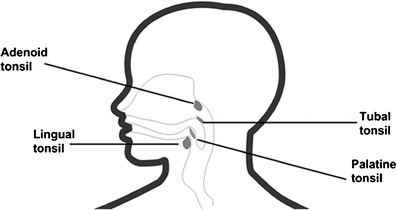
Waldeyer’s ring is an induction site for mucosal responses. It is formed by the lymphoid tissues near the opening of the respiratory and digestive systems. It consists of the adenoid, tubal, palatine and lingual tonsils

Mucosal vaccination can be achieved via a number of routes including oral, intranasal, pulmonary, rectal, or vaginal [6]. Of these, the nasal route is the most straightforward and is suitable for vaccine administration. Advantages and disadvantages of nasal vaccination are summarized in Table 1. The prime inductive site for mucosal immunity in the nasopharyngeal tract in rodents is the nasal-associated lymphoid tissue (NALT) [7–9]. NALT is considered the rodent equivalent of Waldeyer’s ring (Fig. 1), the lymphoid tissues (tonsils) present in humans [10]. Waldeyer’s ring forms a protective site at the opening of the pharynx to provide immunity [11]. Protection at this mucosal surface is correlated with secretory immunoglobulin-A (sIgA) antibodies which, alongside other innate defence mechanisms, provide additional protection from pathogens [12]. Murine NALT is a functional analogue to human tonsils, and animal research has provided insight into human immunology. Such studies are essential before any human trials of vaccine candidates. However, human immunity and biological composition can differ from those of animal models. For nasal vaccine development, development of appropriate in vitro models has provided a reproducible approach in which phenotypic and physiological features of the NALT can be simulated [13]. Techniques to collect and analyse mucosal secretions and cell types have also provided a rational approach for evaluation of vaccine efficacy [14].

**Table 1:** Advantages and disadvantages of intranasal vaccination

Advantages	Disadvantages
Needle-free thus patient compliance	Rapid clearance
Non-invasive	Inefficient uptake
Small antigenic dose	Lack of human compatible mucosal adjuvant
Induction of systemic and mucosal immunity	
Immunity at primary and distal mucosal sites

**Fig. 2:**
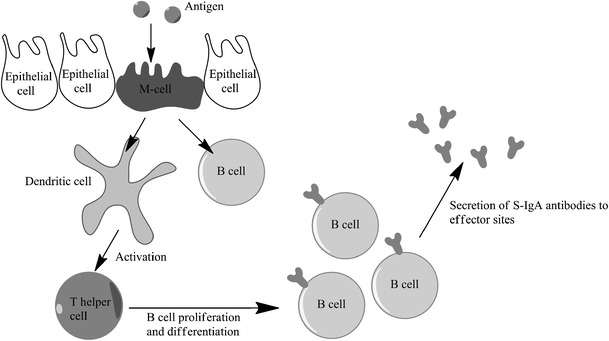
Scheme of immunological pathway for induction of local mucosal response in NALT

NALT comprises a organised structure of cells involved in the induction of an immune response, including dendritic cells, T cells and B cells, which are covered by an epithelial layer of cells containing distinctive cells called M cells [9]. M cells in the NALT are the sites of antigen uptake for induction of mucosal immunity [15]. Whilst small soluble antigens are able to penetrate the nasal epithelium, particulate antigens are mainly taken up by M cells in the NALT [16]. Antigen is actively transported by M cells, to reach dendritic cells, macrophages and B cells, for processing and presentation [17]. Consequent activation of antigen-specific CD4+ T helper cells (Th cells) interact with B cells which develop into IgA committed (IgA+). IgA+ B cells move to effector sites such as the nasal passage where they differentiate into IgA-producing plasma cells and secrete IgA in dimers. Dimeric IgA then become S-IgA by binding to the polymeric Ig receptor which transports IgA to effector sites [18]. An overview of the immune response in NALT is summarized in Fig. 2. S-IgA are able to bind toxins, bacteria or viruses and neutralize their activity, thus preventing entry into the body or reaching the internal organs. Whilst this can prevent infection through the mucosal epithelium, certain pathogens such as group A streptococcus can concurrently infect through the skin. The advantage of vaccination at the mucosal surface by intranasal administration is the induction of mucosal and systemic immune responses, whereas traditional parenteral administration generally only results in systemic immune responses. Therefore, in the context of group A streptococcus and other pathogens which concurrently infect through the mucosa and systemic sites, an ideal vaccine could be an intranasally administered vaccine eliciting neutralizing IgA preventing colonization of the throat and systemic IgG antibodies facilitating clearance from systemic sites [11].

An effective vaccine formulation for intranasal delivery maintains the antigen in a stable form, ensures the antigen, remains in the nasopharyngeal region long enough for the antigen to interact with the lymphatic system and stimulates the immune system—with or without additional adjuvants—to provide long-term immunity [19]. This review discusses the leading strategies in delivery systems and adjuvants currently under investigation for the development of nasal vaccines.

## Attenuated intranasal vaccines

Nasally delivered influenza vaccines based on live-attenuated strains are currently under investigation to prevent seasonal and pandemic influenza. Live-attenuated vaccines have the advantage of mimicking natural infection by presenting influenza antigens in their native conformation to the nasal mucosal tissues, without inducing flu-like symptoms [19]. An example of this type of vaccine is FluMist by Astra-Zeneca, the first nasal trivalent vaccine for seasonal influenza [20, 21]. When compared with an injectable trivalent influenza vaccine, the FluMist vaccine conferred a longer duration of protection, better cross-protection, enhanced efficacy and both mucosal and systemic immunity [19]. Despite the promise of the live-attenuated influenza vaccine strategy, adverse side effects have been reported including safety concerns for patients with allergy or asthma as well as irregular side effects similar to Bell’s palsy [19].

## Enhancement of mucoadhesion

Most antigens have little to no affinity for the nasal epithelium and tend to be removed quickly by mucociliary clearance [22, 23]. Extending the nasal residence time by co-administering the antigen with mucoadhesives is a logical approach to enhance absorption and residence time to facilitate interaction with the immune system [16, 24]. Significant results with biodegradable, mucoadhesive polymeric carriers have highlighted their use for mucosal vaccine delivery. Polymers such as polylactide-co-glycolide (PLGA), chitosan, alginate and carbopol have been explored for the delivery of antigens via the intranasal routes [23]. Hydrophilic polymers, like sodium alginate and carbopol, absorb to the mucus by forming hydrogen bonds, consequently enhancing nasal residence time [25, 26].

Sodium alginate is a linear copolymer and consists of 1–4-linked β-d-mannuronic acid and 1–4-linked α-l-guluronic acid residues. Sodium alginate is biocompatible and mucoadhesive and has been used for controlled delivery of drugs following intranasal administration [27, 28]. In vivo studies showed that the therapeutic efficacy of a drug-sodium alginate formulation significantly improved in comparison to nasal administration of drug alone [27]. Good biocompatibility, low cost and ease of preparation are the major advantages of using alginate polymers for the delivery of an antigen [29]. Extensive use of sodium alginate as an antigen delivery system to improve the efficacy of mucosal immunization in livestock has been carried out [30]. For example, induction of systemic and mucosal immune response in cattle by intranasal administration of antigen in alginate microspheres has been reported [31].

Carbopol is a cross-linked polyacrylic acid polymer [32]. When mixtures of starch and carbopol have been used as carriers of influenza virus antigen, systemic antigen-specific IgG responses were induced after intranasal delivery [33]. The level of IgG and the immune response kinetics were improved by the presence of carbopol [33]. Carbopol has been used experimentally as a delivery system to enhance adhesion to mucosal surfaces and may facilitate enhanced protection of peptides and proteins against enzymatic degradation [34]. Carbopol has received considerable attention delivery of proteins, and Witschi and Mrsny showed (using bovine serum albumin as model antigen) that carbopol gels can be used for nasal protein delivery [35].

Chitosan is a non-toxic linear polysaccharide produced by chitin deacetylation. Chitin is a naturally occurring polymer found in the exoskeletons of insects and crustaceans [36]. As a cationic polymer, chitosan interacts with negatively charged mucin by ionic interactions [37–39]. Mucociliary clearance is decreased, and a transient effect on the paracellular transport of antigens has been observed [40]. In addition to being a mucoadhesive, chitosan has also been reported to have adjuvant properties that enhance humoral and cellular immune responses [41–43] via opening of intercellular tight junctions, which favours the transport of antigens [44]. Combination of transient opening of tight junctions and mucoadhesive properties most likely enables the interaction of the chitosan-antigen formulation to interact with the lymph nodes of the nasal cavity, leading to improved immunological responses [45]. For controlled vaccine release, chitosan nanoparticles are reported to be effectively taken up by antigen presenting cells and induce strong mucosal and systemic immune responses against antigens [36, 46, 47].

Chitosan-based formulations have been used to improve the delivery and immunogenicity of a variety of antigens. Luteinizing hormone-releasing hormone (LHRH) displays poor immunogenicity and requires the presence of an adjuvant. Chitosan-based formulations were studied as a potential adjuvant for a vaccine against LHRH. A reduction in animal steroidogenesis and spermatogenesis was observed when chitosan was used as an adjuvant, showing the ability of this delivery system to neutralize LHRH [48]. The influenza A, matrix protein 1 (M1), is highly conserved and can form the basis of a vaccine. Mice intranasally administered M1 and chitosan were challenged with lethal doses of H9N2, H1N1 and H5N1 viruses [46]. Effective cross-protection against influenza virus was observed for the vaccine candidate [35]. The results showed chitosan had an improvement over the efficacy of the M1-based vaccine alone [46]. Additionally, nasal immunization with *N*-trimethyl chitosan (TMC)-based nanoparticles increased the nasal residence time of ovalbumin, a model antigen. After intra-mucosal administration, slow antigen-releasing TMC nanoparticles did not induce detectable antibody titres whereas fast antigen-releasing TMC nanoparticles showed high sIgA levels and serum antibody titres [49]. It was found that these nanoparticles were mucoadhesive and stimulated the maturation of dendritic cells [49]. Furthermore, a formulation of chitosan and DNA encoding a viral protein from coxsackievirus B3 was shown to induce high levels of mucosal sIgA and serum IgG [50]. Following intranasal challenge with lethal CVB3, significant reduction of viral load was observed [50]. Chitosan was reported to slow down the nasal mucociliary clearance and prolong the contact period between the NALT and the chitosan–antigen complex [50, 51]. These examples with chitosan illustrate that the co-administration of antigens with mucoadhesive agents can enhance efficacy, potentially reduce antigen dose and generally facilitate the development of mucosal vaccine delivery systems [52]. Since most vaccines alone are not sufficiently taken up after mucosal administration, co-administered with penetration enhancers, adjuvants or encapsulation in particles are various approaches to overcome this [23]. Immunogenicity enhancing properties of chitosan due to combination of transient opening of tight junctions and mucoadhesive properties makes it promising for mucosal vaccine delivery.

## Particulate delivery systems

Particulate delivery systems used for nasal administration of vaccines include liposomes, immune-stimulating complexes (ISCOMs) and polymeric particles—including virosomes [19, 53–55]. Particulate antigens use the transcellular route to reach the lymphoid tissues and target M cells. M cells, which are part of the NALT, act as portals of entry for antigens to enter regions containing professional antigen presenting cells (dendritic cells), B cells and T cells, thereby contributing to both humoral and cellular immune responses [15, 17, 56, 57]. Particulate systems have the capacity to present multiple copies of the antigen and tend to have a similar size to pathogens, mimicking natural infection.

Liposomes are particulate vesicles composed of different ratios of lipids and cholesterol enclosing an aqueous core, enabling the incorporation of a wide variety of antigens. The immunogenicity conferred by liposomes is due to: their ability to accommodate multiple copies of antigens, preferential uptake by macrophages, protection within the biological environment and effects on the intracellular processing of antigen [19, 58]. Surface-modified (glycol chitosan or oligomannose coated) liposomes have been shown to elicit humoral and cellular immune responses that were significant (when compared to antigen alone) following intranasal administration [59, 60]. Surface charge tends to influence the immunogenicity of mucosal liposomal vaccine formulations. Fusogenic and cationic–fusogenic liposomes encapsulated with antigens have been demonstrated to effectively stimulate a mucosal immune response [61].

ISCOMs are made up of saponin, as an adjuvant, along with lipids and an antigen, and are generally held together by hydrophobic interaction between the constituents. The essential components to form ISCOMs are cholesterol and saponin [62]. Their inherent particulate nature, multimeric antigen presentation and potent immunostimulatory activity make ISCOMs an attractive choice for vaccine delivery [19, 63–65]. Several studies have shown that ISCOM-incorporated antigens induce specific local and systemic immune responses following intranasal administration, conferring protection against influenza, respiratory syncytial virus and hepatitis B [66–69].

Virosomes are an alternative particulate delivery form composed of extracted glycoproteins from virus particles and mimic viral structures. The viral glycoproteins present on virosomes have a high affinity for the mucosal surfaces of the respiratory tract [70]. Virosomes enable efficient induction of humoral and cellular responses and target dendritic cells [71–76]. They have been demonstrated to be efficient nasal delivery systems for several antigens, including DNA [75], and influenza [77, 78] and HIV proteins [79].

Particulate systems are promising for the nasal delivery of vaccines, enhancing uptake by antigen-presenting cells, conferring a depot effect and protecting the antigen from degradation. Further research in the physicochemical properties of particulates that influence immunogenicity will contribute to the development of nasal vaccine delivery systems.

## Lipopeptide-based delivery systems

**Fig. 3:**
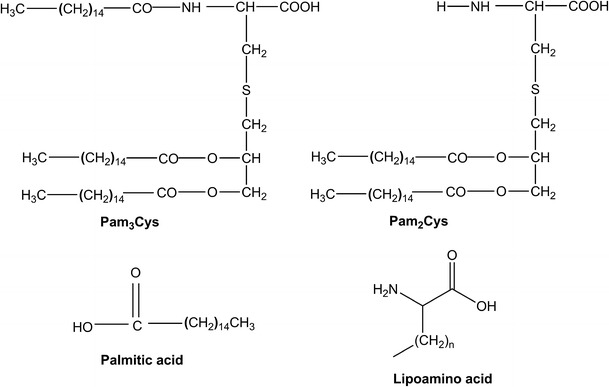
The structure of the lipid moieties used to enhance immunogenicity of weak antigens

Lipopeptides of bacterial origin, or their synthetic derivatives, represent potent immunostimulants when given in combination with peptide or protein antigens [80]. Examples of lipid moieties include tri-palmitoyl-*S*-glyceryl cysteine (Pam3Cys), di-palmitoyl-*S*-glyceryl cysteine (Pam2Cys), single/multiple-chain palmitic acids and lipoamino acids (LAAs) (Fig. 3). The desirable immunological activity of these lipid moiety conjugates arises from their intrinsic adjuvant properties, such as B cell and macrophage activators, ability to induce maturation of dendritic cells and promote an inflammatory response [81–85]. Generally, this occurs via signalling through receptors of the immune system that recognise these lipid moieties as pathogen-associated molecular patterns [86–89].

Lipopeptides containing Pam3Cys and Pam2Cys have been evaluated for development of immunocontraceptive vaccines. When administered intranasally, the constructs were highly immunogenic, capable of inducing high titres of antibodies and efficiently sterilized female mice when administered alone in saline [90]. A Pam2Cys-based vaccine against *Streptococcus pyogenes* had the ability to induce mucosal and systemic antibodies following intranasal immunization and conferred protection against bacterial challenge [91]. For influenza, Pam2Cys-based lipopeptides have been shown to confer protection by inducing a long-term cellular response [92].

Lipopeptide vaccines based on a palmitic acid moiety may present an ideal strategy against pathogens that infect mucosal surfaces. Intranasal administration of a palmitoylated lipopeptide vaccine for human cytomegalovirus (a herpes virus) elicited both systemic and local mucosal cellular responses when administered intranasally [93]. Herpes simplex virus type 2 peptide epitopes conjugated to a palmitic acid moiety have shown the ability to prevent transmission and/or limit the severity of diseases [94].

**Fig. 4:**
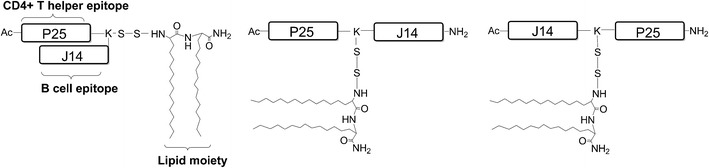
Structures of lipopeptides with a T helper epitope (P25), *S. pyogenes* B cell epitope (J14) and a lipid moiety based on LAAs

A library of lipopeptide vaccine candidates composed of a *S. pyogenes* B cell epitope, a T helper epitope and a lipid moiety based on LAAs (Fig. 3) has also been investigated (Fig. 4). The orientation of each component of the lipopeptide was optimized to elicit a strong immune response following intranasal immunization. The antibody titres elicited in response to these lipopeptides provided important information for the design of an effective, lipopeptide-based mucosal vaccine [95, 96]. Interestingly, the lipopeptides were self-adjuvanting, negating the use of additional adjuvants, which could easily be applied to other peptide-based vaccines. An important consideration for lipopeptide vaccines is ensuring that the lipid moiety is easy to synthesize, couple to the antigen, purify and formulate. As a result, LAAs are an attractive option because they can be synthesized and coupled to peptide antigens using standard peptide synthesis methods [97].

Lipopeptides provide several advantages for nasal vaccine development. Firstly, they negate the requirement for adjuvant, which has the potential to induce adverse side effects. Secondly, lipopeptide vaccine candidates can induce cellular and antibody responses, allowing control over the desired immune response. Current pre-clinical and clinical trials with lipopeptides suggest they are effective, non-toxic and can be synthesized using current methods of peptide synthesis to a high purity and yield. The lipopeptide-based approach for the nasal delivery of vaccines induces effective mucosal and systemic immune responses [82].

## DNA-based vaccines

DNA vaccines have been investigated for intranasal mucosal vaccine development. DNA-based vaccines where antibodies raised to specific proteins involved in immunity to infection have shown promise in animal models. The composition and negative charge of DNA generally hinders the entrapment efficiency and stability of vaccine formulations. Several investigators have used cationic components such as chitosan and polyethylenimine (PEI) to complex with DNA antigens in the form of nano-/microparticles for intranasal delivery [19]. Plasmid DNA containing chitosan nanoparticles for nasal immunization against nucleocapsid (N) protein of severe acute respiratory syndrome coronavirus (SARS-CoV) showed elicitation of mucosal IgA as well as systemic IgG against N protein [98]. SARS DNA vaccine complexed with other cationic polymers effectively delivered the plasmid DNA to induce antigen-specific humoral and cellular immune responses [99]. PEI is of particular interest for intranasal delivery due to reported many-fold increase in gene transfer in the respiratory tract. Using PEI formulation, intranasal vaccination with DNA-encoding influenza A H5N1 or H1N1 antigens, high levels of antibodies were detected in bronchoalveolar lavages and the serum [100].

Cationic PLGA particles have also been used as an intranasal delivery system for administering foot and mouth disease (FMDV) vaccine encoding the FMDV capsid protein [101]. Intranasal delivery of the cationic PLGA particles containing FMDV DNA vaccine formulations enhanced protective immunity against FMDV [101]. Whilst DNA vaccines have been shown promise for intranasal vaccine delivery, the technology requires further research to develop effective vaccines [19].

## Mucosal adjuvants

Improving the immunogenicity of antigens through the use of adjuvants (which can also act as delivery systems) is a rational approach for vaccine development. Nevertheless, achieving a potent adjuvant effect whilst avoiding reactogenicity or toxicity is a major challenge. Peptide and protein antigens suffer from poor immunogenicity and require the use of adjuvants. However, existing licensed adjuvants, such as alum and to a lesser extent MF59, are not suitable as adjuvants for mucosal vaccine administration and in general do not induce mucosal antibodies [102]. Unlike the systemic immune system, mucosal surfaces regularly encounter an extensive range of foreign material. To accommodate this, the mucosal immune system must be selective in responding to antigens in order to avoid undesirable immune responses and excessive activation of the immune system [103]. Thus, induction of mucosal immunity is more difficult, and novel strategies are critical to the successful development of mucosal adjuvants.

Dendritic cells and M cells are the major cell types to be targeted by a mucosal adjuvant. CpG oligodeoxynucleotides (CpG ODN) Flt3 ligand and monophosphoryl lipid A (MLA) represent dendritic cell targeting ligands [104], whilst Flt3 is a growth factor reported to stimulate dendritic cells [105]. CpG ODN mimics the immunostimulatory effects of bacterial DNA and is known to target plasmacytoid dendritic cells for their activation, maturation [106]. Mucosal administration of CpG ODN with model antigens, including influenza virus and tetanus toxoid, effectively elicited antigen-specific immunity [107, 108].

Furthermore, Flt3 has been demonstrated as a safe adjuvant with nasal dendritic cell targeting properties that confer protection against fatal pneumococcal pneumonia in mice [109]. To this end, CpG ODN and Flt3 represent mucosal dendritic cell targeting adjuvants for the induction of antigen-specific, protective mucosal immune responses.

**Fig. 5:**
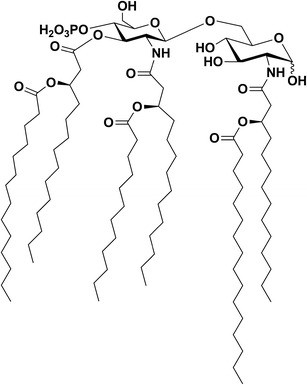
Chemical structure of MLA

Bacterial lipopolysaccharide is a potent immunostimulatory agent but exhibits extreme toxicity [110]. Chemical modifications to alleviate its toxic effects resulted in the identification of MLA (Fig. 5) [111]. The clinical grade MLA formulation corresponds to the main active component of Corixa’s MPL adjuvant [111]. In numerous preclinical and clinical studies, MPL has proven to be a potent yet non-toxic adjuvant. It has been used extensively as an adjuvant in human vaccine trials for several infectious diseases and cancer [111]. Effectiveness of MPL as a mucosal adjuvant was investigated following intranasal administration of a formulation of MPL added to soluble antigen or liposomes encapsulating antigen [112]. The liposomal antigen formulation with MPL resulted in IgA responses that were consistently higher than seen in mice immunized with liposome antigen or free antigen without MPL [112]. These results demonstrated the effectiveness of MPL as an adjuvant for potentiating mucosal immune responses.

M cells provide a portal of entry for pathogens, through which they can invade the host. Identification of the molecules that are required for bacterial or viral entry through M cells may provide a rational basis for developing an effective mucosal adjuvant/delivery system [104]. For example, reoviruses initially infect through M cells using their surface protein sigma-1 (pσ1) [113]. An M cell-targeting DNA vaccine formulation consisting of plasmid DNA and the reovirus pσ1 induced significant mucosal and systemic immunity [114]. This suggests that M cell targeting could be a useful approach for the development of a mucosal vaccine.

The above examples represent a sample of the strategies that could be used to facilitate the development of novel mucosal adjuvants. However, mucosal adjuvants must still overcome the two major hurdles: effectiveness and safety. These are both relatively more difficult objectives compared with the development of vaccines that elicit systemic immunity, due to the uniqueness and complexity of the mucosal immune system.

## Conclusion

The development of novel, mucosally active, intranasally administered vaccines has the potential to provide immunity against a myriad of infectious diseases. Mucosal administration of vaccines presents an ideal strategy against many pathogens that infect via mucosal surfaces. A suitable combination of adjuvants targeting M cells or dendritic cells and delivery systems that are mucoadhesive could facilitate the development of effective nasal vaccines. The use of the non-invasive, needle-free nasal route is advantageous for vaccination programmes, since it can enhance patient compliance and reduce need to be administered by specialised healthcare workers.
